# Relevant Spatial Scales of Chemical Variation in *Aplysina aerophoba*

**DOI:** 10.3390/md9122499

**Published:** 2011-11-28

**Authors:** Oriol Sacristan-Soriano, Bernard Banaigs, Mikel A. Becerro

**Affiliations:** 1 Center for Advanced Studies of Blanes (CEAB-CSIC), Accés a la Cala St. Francesc 14, Blanes 17300, Girona, Spain; Email: osacristan@ceab.csic.es; 2 Environmental and Biomolecular Chemistry Laboratory, University of Perpignan Via Domitia, 52 Paul Alduy Ave., Perpignan Cedex 66860, France; Email: banaigs@univ-perp.fr; 3 Natural Products and Agrobiology Institute (IPNA-CSIC), Avda. Astrofísico Francisco Sánchez 3, La Laguna, Tenerife 38206, Spain

**Keywords:** brominated alkaloids, geographic variation, natural products, Porifera, sponges, secondary metabolites

## Abstract

Understanding the scale at which natural products vary the most is critical because it sheds light on the type of factors that regulate their production. The sponge *Aplysina aerophoba* is a common Mediterranean sponge inhabiting shallow waters in the Mediterranean and its area of influence in Atlantic Ocean. This species contains large concentrations of brominated alkaloids (BAs) that play a number of ecological roles in nature. Our research investigates the ecological variation in BAs of *A. aerophoba* from a scale of hundred of meters to thousand kilometers. We used a nested design to sample sponges from two geographically distinct regions (Canary Islands and Mediterranean, over 2500 km), with two zones within each region (less than 50 km), two locations within each zone (less than 5 km), and two sites within each location (less than 500 m). We used high-performance liquid chromatography to quantify multiple BAs and a spectrophotometer to quantify chlorophyll *a* (Chl *a*). Our results show a striking degree of variation in both natural products and Chl *a* content. Significant variation in Chl *a* content occurred at the largest and smallest geographic scales. The variation patterns of BAs also occurred at the largest and smallest scales, but varied depending on which BA was analyzed. Concentrations of Chl *a* and isofistularin-3 were negatively correlated, suggesting that symbionts may impact the concentration of some of these compounds. Our results underline the complex control of the production of secondary metabolites, with factors acting at both small and large geographic scales affecting the production of multiple secondary metabolites.

## 1. Introduction

Conducting research at multiple spatial or temporal scales can substantially increase our understanding of numerous ecological processes, making scale a central problem in ecology [1]. Ecological processes and the mechanisms behind them show large spatial and temporal variation, which results in a high degree of heterogeneity across wide ranges of space, time, and biological organization (e.g., from ecosystems, to species, to species traits). Moreover, a multiple scale approach could shed light on the factors behind natural variation, improve our understanding of the ecological role of specific traits, and show evidence of the relevance or universality of ecological mechanisms and processes [1]. Besides, there is no single natural scale at which ecological studies should be studied [1]. Yet, ecological studies over large spatial and temporal scales are often hard to carry out and are uncommon on numerous ecological areas.

The production of marine natural products is an area that could benefit tremendously from a multi scale approach. We know that marine natural products play multiple roles in nature [2,3,4] and vary remarkably as a function of space and time [5,6,7]. The factors behind such variation are far from being fully understood and this research area is in chronic need of empirical data [8,9]. In fact, the poor understanding of the processes that control chemical diversity and variation is hindering the development of marine chemical ecology [9]. It is therefore critical to investigate chemical variation because it will shed light on the factors that regulate the production of chemical defenses, building up the field of marine chemical ecology.

The production of natural products is widespread in the benthic realm [10]. Sponges are consistently the richest source of marine natural products [10,11] and have also received considerable attention from a chemical ecology perspective [9,12]. Sponge secondary chemistry is known to vary significantly within species as a function of time [13,14,15,16], geographic region [13,17], habitat/community [18,19,20], specimens [21,22], tissues [23,24,25], and cells [26,27]. In particular, there is abundant information on secondary chemistry and chemical ecology of the genus *Aplysina*. There are multiple bromotyrosine alkaloids (BAs) described for *Aplysina* spp. [28,29,30,31,32], which can represent up to 13% of the sponge dry mass [33]. These compounds have a variety of biological activities [34,35,36,37] and play multiple ecological roles in nature [38,39,40]. Changes in the chemical structure of these compounds alter their biological activity [39,40], so the exact composition and concentration of compounds in the sponge tissues could translate into differential ecological roles. 

BAs may vary remarkably within the same *Aplysina* species [41], which could have important ecological implications. The BAs of *Aplysina aerophoba* vary between cells [27], tissues [24,42,43], specimens [43,44], and geographic location [33]. Moreover, this species is distributed in the Mediterranean and Canary islands, where it can be locally abundant [33,42]. These characteristics make *Aplysina aerophoba* a perfect organism to investigate natural product variation across multiple spatial scales ranging from a few meters to thousands of kilometers apart, and thus provide a great opportunity to assess the spatial scale at which chemical variation varies the most.

It is known that photosynthetic symbionts can contribute to the production of secondary metabolites [25,45,46] and might be involved somehow in the secondary chemistry of *A. aerophoba* [24,47]. Since concentration of Chlorophyll *a* (Chl *a*) can be used as a traditional proxy of the abundance of photosynthetic symbionts in sponges [23,42,48,49], we assessed whether the concentration of Chl *a* varied at the same spatial scale as BAs and further investigated whether both Chl *a* and secondary metabolites were related. 

Is there a spatial pattern of variation in BAs? Do BAs vary the most between, near, medium, or far away locations? Do individual BAs follow the same pattern of variation? Do these patterns match the pattern of variation in Chl *a*? In this study we tested these hypotheses by looking at the spatial variation in BAs and Chl *a* of the sponge *Aplysina aerophoba* in neighboring bays to locations over 2500 km apart. We followed a nested design with two distant geographic regions, two zones within each region, two locations within each zone, and two sites within each location. This design calculates the magnitude of the variance attributable to the four spatial scales and will suggest the most relevant scale at which chemical variation should be addressed in this species. We found a spatial scale where BAs and Chl *a* varied the most and shed light on the complex mechanisms behind the production of natural products within a single species. This is the first report to take a broad spatial approach in marine chemical ecology, but further research will clarify whether this complex trend in the production of natural products is common among benthic organisms. 

## 2. Results and Discussion

### 2.1. Natural Product and Chlorophyll *a* Quantification

**Figure 1:**
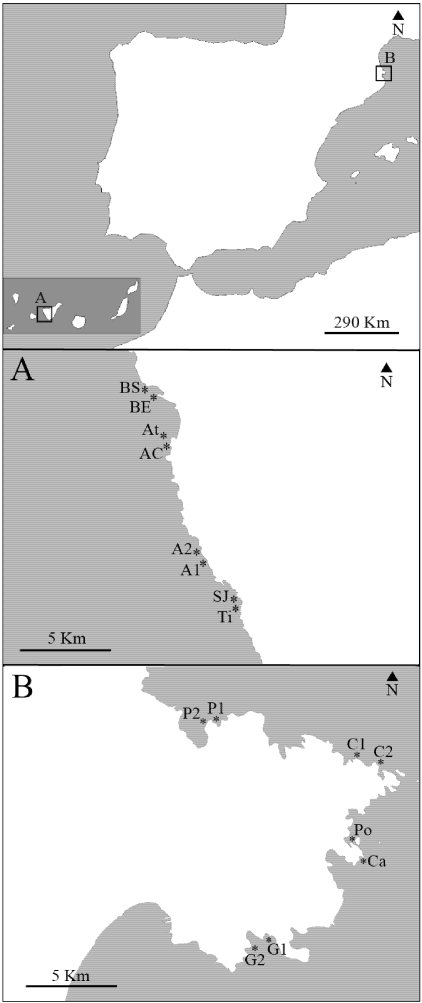
Study area that comprises two biogeographic regions; Tenerife (**A**; Canary Islands) and Cap de Creus (**B**; Northwestern Mediterranean). (**A**) Sampling sites of Tenerife. Punta Tixera (Ti), San Juan (SJ), Alcalá 1 (A1), Alcalá 2 (A2), Atlántida Coast (AC), Atlántida (At), Barranco del Eco (BE), Barranco Seco (BS); (**B**) Sampling sites of Cap de Creus. Gat 1 (G1), Gat 2 (G2), Caials (Ca), Portlligat (Po), Club Med 1 (C1), Club Med 2 (C2), Port de la Selva 1 (P1), Port de la Selva 2 (P2).

We quantified a total of 126 samples (2 regions × 2 zones per region × 2 locations per zone × 2 sampling sites per location × 6–10 individual sponges per sampling site; Figure 1) to characterize the chemical profile and determine the Chlorophyll *a* concentration (Chl *a*) of *Aplysina aerophoba*. We identified and quantified the four major brominated alkaloids in our samples: aerophobin-1 (Aero1; peak 3), aerophobin-2 (Aero2; peak 4), aplysinamisin-1 (Aply1; peak 5), and isofistularin-3 (Iso3; peak 6), by comparing their retention times and UV profiles to those of purified, characterized standard compounds. 

The secondary metabolites identified were in agreement with the abundant literature available for this species [30,39,40,44]. To minimize compound degradation and the enzymatic transformation of the high molecular weight (HMW) BAs quantified in our study into the low molecular weight (LMW) natural products reported elsewhere [36,39,50,51], we used a sampling protocol that minimized manipulation of live tissues, froze our samples rapidly after collection, and used methanol alone to obtain crude extracts. Since these LMW natural products result from bioconversion of precursors (but see [22]), their presence in our chromatograms could cast doubt on the actual concentration of the precursors quantified in our samples [39]. We failed to observe these bioconverted natural products in our chromatograms or they were at such low concentrations that cannot explain the large variation in BAs observed in our samples (see below). The concentrations of the four compounds we quantified were positively correlated, except for the negative correlation between Aerophobin-2 and Aplysinamisin-1 (Table 1). Although this negative correlation could be indicative of bioconversion between these two compounds (concentration of a precursor decreases as concentration of the resulting compound increases), the degradation of Aerophobin-2 and Isofistularin-3 results in the LMW compounds [37], which were undetected in our extracts. 

**Table 1:** Correlation coefficients (below diagonal) and uncorrected *P*-values (above diagonal) between depth and the concentrations of chlorophyll *a* (Chl *a*), Aerophobin-1 (Aero1), Aerophobin-2 (Aero2), Aplysinamisin-1 (Aply1), Isofistularin-3 (Iso3), and the four BAs combined (All).

	**Depth**	**Chl *a***	**All**	**Aero1**	**Aero2**	**Aply1**	**Iso3**
**Depth**		<0.001 *	0.001 *	0.008	0.276	0.494	<0.001 *
**Chl *a***	−0.391 *		0.001 *	0.059	0.459	0.796	<0.001 *
**All**	0.294 *	−0.283 *		---	---	---	---
**Aero1**	0.236	−0.168	---		<0.001 *	<0.001 *	<0.001 *
**Aero2**	0.098	−0.067	---	0.403 *		<0.001 *	0.001 *
**Aply1**	0.062	−0.023	---	0.543 *	−0.466 *		0.508
**Iso3**	0.349 *	−0.430 *	---	0.385 *	0.294 *	0.060	

* show significant correlation coefficients and *P*-values after Bonferroni correction; --- correlation between the four individual compounds and their combined concentration are meaningless and not reported.

### 2.2. Secondary Metabolite Variation

The total concentration of BAs varied between sampling sites but not between regions, zones, or locations (nested analysis of covariance, ANCOVA, *P* < 0.001; *P* = 0.722; *P* = 0.086; and *P* = 0.650, respectively, Table 2). Because we found that depth could explain 8.6% of the variability in the total concentration of BAs (*R*^2^ = 0.086, Table 1) we used depth as a covariate in our analyses. Total concentration of BAs did not vary with depth (Table 2), but 28.41% of the total variance in BAs occurred between sites that are less than 500 m apart (Table 2).

**Table 2:** *P*-values and percentage of the total variance explained by depth, region, zone, location, and site. Values obtained from nested ANCOVAs used to test the effect of spatial scale on the concentration of chlorophyll *a*, Aerophobin-1, Aerophobin-2, Aplysinamisin-1, Isofistularin-3, and total concentration of BAs with Depth as covariate. We also show the percent variance unexplained by the factors (Error). Geographic scale included Region (over 2500 km), Zone (less than 50 km), Location (less than 5 km), and Site (less than 500 m).

Compound	Depth	Region	Zone	Location	Site	Error
Chlorophyll *a*	0.069	<0.001/31.76%	0.905	0.236	0.038/9.46%	59.33%
Total BAs	0.275	0.722	0.086	0.650	<0.001/28.41%	68.58%
Aerophobin-1	0.382	0.762	0.054	0.694	<0.001/21.95%	53.40%
Aerophobin-2	0.064	0.945	0.060	0.736	0.001/28.47%	72.27%
Aplysinamisin-1	0.595	<0.001/18.34%	0.955	0.102	0.273	76.26%
Isofistularin-3	0.937	0.012/46.27%	0.555	0.388	0.110	49.20%

**Figure 2:**
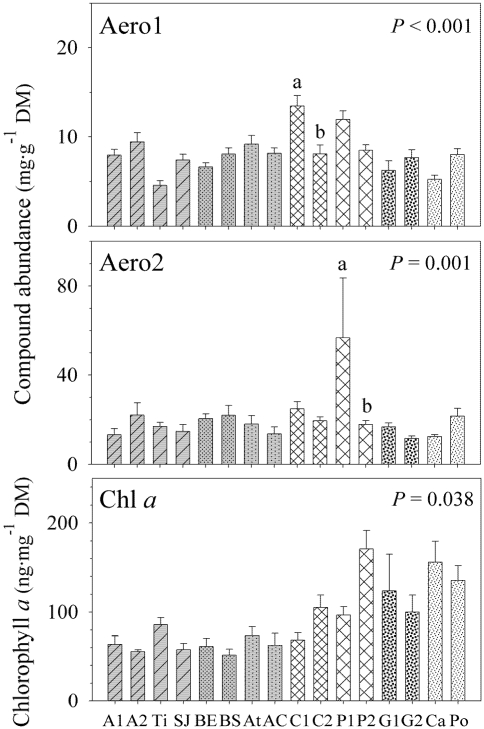
Significant differences in Aerophobin-1, Aerophobin-2 (mg·g^−1^ dry mass sponge tissue ± 1 SE), and Chlorophyll *a* concentrations (ng·mg^−1^ dry mass sponge tissue ± 1 SE) of *Aplysina aerophoba* between sampling sites. A1 = Alcalá 1 (*N* = 6); A2 = Alcalá 2 (*N* = 7); Ti = Punta Tixera (*N* = 7); SJ = San Juan (*N* = 9); BE = Barranco del Eco (*N* = 8); BS = Barranco Seco (*N* = 7); At = Atlántida (*N* = 6); AC = Atlántida Coast (*N* = 7); C1 = Club Med 1 (*N* = 10); C2 = Club Med 2 (*N* = 10); P1 = Port de la Selva 1 (*N* = 10); P2 = Port de la Selva 2 (*N* = 10); G1 = Gat 1 (*N* = 6); G2 = Gat 2 (*N* = 8); Ca = Caials (*N* = 7);Po = Portlligat (*N* = 8). Aero1 = aerophobin-1; Aero2 = aerophobin-2; Chl *a* = chlorophyll *a*. Letters indicate significant differences (*P* ≤ 0.05) of pairwise comparisons between sampling sites of the same location (showed as the same pattern design).

**Figure 3:**
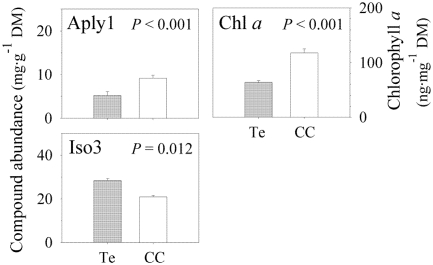
Significant differences in Aplysinamisin-1, Isofistularin-3 (mg·g^−1^ dry mass sponge tissue ± 1 SE), and Chlorophyll *a* concentrations (ng·mg^−1^ dry mass sponge tissue ± 1 SE) of *Aplysina aerophoba* between Tenerife (Te, *N* = 57) and Cap de Creus (CC, *N* = 69). Aply1 = aplysinamisin-1; Iso3 = isofistularin-3; Chl *a* = chlorophyll *a*.

Separate nested ANCOVAs on the concentrations of each of the four compounds showed the relevance of spatial scale in the production of these compounds. The abundance of Aerophobin-1 and Aerophobin-2 differed significantly at the lowest scale (sampling site; *P* < 0.001 for both compounds; Figure 2 and Table 2) while the concentrations of Aplysinamisin-1 and Isofistularin-3 did not vary at this scale (Aply1, *P* = 0.273; Iso3, *P* = 0.110, Table 2). This pattern reversed at the largest geographic scale investigated in our study (Region, over 2500 km apart). The compounds that did not vary between sampling sites, varied significantly across regions (over 2500 km apart) and *vice versa* (region; Aply1, *P* < 0.001; Iso3, *P* = 0.012; Aero1, *P* = 0.762; Aero2, *P* = 0.945; Figure 3 and Table 2). Intermediate scales were nonsignificant for all compounds, although zone was almost significant for Aerophobin-1 and Aerophobin-2 (Table 2). Thus, Region explained 18.34% and 46.27% of the total variance in Aplysinamisin-1 and Isofistularin-3 while Sampling Site explained 21.95% and 28.47% of the total variance of Aerophobin-1 and Aerophobin-2 (Table 2). Depth failed to explain the concentration of any of the four BAs investigated (Table 2).

An earlier study on the concentration of BAs in *Aplysina aerophoba* around the Canary Islands showed that the concentration of BAs varied spatially, despite the striking chemical similarity in the secondary chemistry of this species in locations over 500 km apart [33]. Our study suggests that the secondary chemistry of *A. aerophoba* varies over small and large spatial scales and our nested design allowed us to quantify the geographic scale that explained the largest variation in the concentration of BAs [52]. We found that the largest variation in the total concentration of BAs occurs at the lowest geographic scale investigated, *i.e.*, specimens less than 500 m apart showed the largest differences in the total concentration of BAs. This pattern was also observed in the compounds Aerophobin-1 and Aerophobin-2, which varied the most at low spatial scales, while Aplysinamisin-1 and Isofistularin-3 varied the most across broad spatial scales. 

Our current knowledge of the production of BAs is incomplete, but it seems reasonable to argue that multiple factors acting at multiple spatial scales determine the concentration of these compounds. Studies from multiple disciplines often report increasing diversities with distance [53,54,55] but changes in sponge secondary chemistry occur at wide range of distances [13,22,56]. A study on the chemical diversity of the Atlanto-Mediterranean *Spongia lamella* showed two types of compounds [17]. One group remained constant among 9 populations scattered along the western Mediterranean and Atlantic coast of Portugal (half the distance of our study) while the other group varied significantly across this scale [17]. The two aerophobin compounds seemed to be affected by factors acting at small spatial scale, whereas Aplysinamisin-1 and Isofistularin-3 varied significantly across large spatial scales. Isofistularin-3 and Aerophobin-2 provide effective chemical defense against predators [40] and other BAs present in *A. aerophoba* show strong cytotoxic, algicide, and antibacterial activities [35,37]. Geographic differences in predation, fouling, competition, or symbionts (see below) among other factors could account for the contrasting patterns of variation of BAs in *A. aerophoba* [13,15,17,19,56]. 

Alternatively, our data could be interpreted as an intriguing evidence of the chaotic nature of chemical variability. This chaotic vision could be sustained by true variation in BAs, or could be the result of multiple factors (e.g., sample manipulation, compound quantification, data analyses). Experimental errors, however, would either amplify or reduce our perception of the existing natural variation. Since our methods minimized compound alteration in this species and were consistent across all samples, we believe that our study accurately describes the natural variation in the production of BAs in *A. aerophoba* from local to regional geographic scale. Although a significant percentage of the variation in BAs in *A. aerophoba* was explained (up to 47%), between 49% and 77% remained unexplained (Table 2); thus it is clear that other, currently unknown, factors account for such variability. Further analyses and understanding of the production of natural products will shed light on this area.

### 2.3. Chlorophyll *a* Variation

Chl *a* slightly decreased with increasing depth, which explained over 15.29% of the variation in Chl *a* in our samples (Table 1). However, the effect of spatial scale was larger than depth (Table 2). Our results showed that 31.76% of the variance in Chl *a* occurred between the two distant geographic regions and 9.46% between sites less than 500 m apart (Table 2). The pattern of Chl *a* variation mirrored the pattern of BAs variation. Chl *a*, Aplysinamisin-1, and Isofistularin-3 varied the most between the two biogeographic regions 2500 km apart. Total concentration of BAs, Aerophobin-1, and Aerophobin-2 varied the most between sites less than 500 m apart, which was the second source of variation in Chl *a*. Because concentration of Chl *a* can be used as a proxy of the abundance of photosynthetic symbionts in sponges [23,42,48] and photosynthetic symbionts can contribute to the production of secondary metabolites [25,45,46] (including *Chloroflexi* photosynthetic bacteria in *A. aerophoba* [24,47]), we further investigated whether the concentration of Chl *a* and secondary metabolites was related.

### 2.4. Relationship Between Natural Products and Chlorophyll *a*

**Figure 4:**
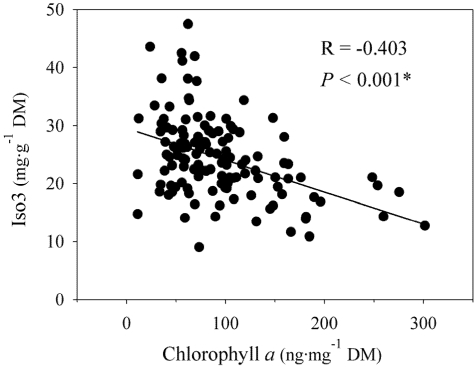
Relationship between the abundance of isofistularin-3 (Iso3; mg·g^−1^ dry mass sponge tissue ± 1 SE) and the concentration of chlorophyll *a* (Chl *a*; ng·mg^−1^ dry mass sponge tissue ± 1 SE). * Significant *P*-value after Bonferroni correction.

Individual correlation analysis resulted in one significant negative correlation between the abundance of Isofistularin-3 and the concentration of Chl *a* (*R* = −0.430, *P* < 0.001; Figure 4, Table 1). We detected no further relationships between Chl *a* and remaining compounds (Table 1). 

Chl *a* in *Aplysina aerophoba* may come from multiple symbiotic sources including photosynthetic cyanobacteria and *Chloroflexi* [24]. Our results suggested that the higher the abundance of photosynthetic symbionts in the sponge, the lower the concentration of Isofistularin-3. Whether this negative association represents a direct relationship between this compound and any of the photosynthetic symbionts in *A. aerophoba* is unclear, but Isofistularin-3 has been shown to be positively associated with the abundance of a symbiotic bacterium [24]. Whether this bacterium decreases its abundance with increasing photosymbiont concentrations remains unknown, but these correlations may reflect a chemically mediated biotic interaction between the sponge host and potential symbionts or between two members of the symbiotic microbial community within *A. aerophoba*.

The diversity of symbiotic communities and natural products found in marine invertebrates is a challenge to assess the role of the symbionts in the production of the compounds typically assigned to the hosts. This is an area that clearly needs further research. *Aplysina aerophoba* has been studied extensively, but we still know very little about factors shaping the production of BAs in this species. Although the BAs seem to be stored in sponge cells [27], multiple cell components might be involved in their production and the role of symbionts is largely unknown [24,43,47,50]. This hypothesis was launched over a decade ago but it remains to be experimentally tested. The advent of new biotechnological methods and approaches will surely shed light on the role of symbionts in the production of natural products [24,57,58].

## 3. Experimental Section

In March 2003, the sponge *Aplysina aerophoba* (Nardo, 1843) was collected by scuba diving at different sampling sites of Tenerife (Canary Islands) and Cap de Creus (Northwestern Mediterranean) (Figure 1). We collected several specimens from each location to obtain enough material for the bulk chemical extraction necessary to set up the chemical methods. To assess a geographic variation in natural products, we used a nested design covering a range of biogeographic scales to collect the whole chimney-like structures representative of this species, which are known to have the same secondary chemistry [24], of about 10 specimens in each site. We used a sharp knife to cut chimneys off by the base and placed them in independent plastic bags. Immediately after collection, samples were placed in coolers with ice to prevent changes in the secondary chemistry and all samples were frozen at −20 °C within 4 h of collection. Once in the laboratory, samples were freeze-dried under dark and a small portion of the top half of the chimney away from the cutting surface was selected for the quantification of the BAs. There are no differences in secondary chemistry between the top and bottom zones of the chimneys [24].

For BA isolation, 50 g of freeze-dried sponge were extracted three times (1 h, 1 h, and overnight) with methanol (MeOH, 20 mL MeOH per 1 g sponge). The crude extract (CE) was first fractionated by Flash-Chromatography. High Performance Liquid Chromatography (HPLC) was performed on a Waters HPLC with an Alliance separation module 2695, column heater, and 2998 photodiode array detector. Separation was achieved using a hydrophobic column (Phenomenex Gemini C18, 110 Å, 250 × 10.0 mm, 5 μm). Mobile phase consisted of 70% of MeOH, 30% of MilliQ water; the flow rate was 3 mL·min^−1^ and the injection volume was 100 µL. Peaks were detected at 320 nm and each BA compound detected was identified and carefully isolated. Dry pure BAs were recovered using rotatory evaporation for use as standards. Then, series of dilution on pure compounds coupled to peak area calculation in HPLC (at 245 nm) allowed tracing calibration-curves. The major compounds observed in the HPLC chromatograms were characterized by classic spectrometric techniques (Liquid Chromatography/Mass Spectrometry, Nuclear Magnetic Resonance, UV profile) and by their retention times. 

For BA quantification, approximately 50 mg of freeze-dried sponge tissue was extracted three times with 1.5 mL of MeOH in an ultrasonic tank for 15 min each time. The CE was filtered through a 20 μm polytetrafluoroethylene filter (PTFE) and added in a 5 mL beaker. The final volume was adjusted to 5 mL with MeOH and an aliquot of 1.5 mL was passed through a 13 mm, 0.2 μm PTFE syringe-filter before HPLC injection. Separation was achieved using a hydrophobic column (Phenomenex Synergi Max-RP, 80 Å, 250 × 3.0 mm, 4 μm) with a mobile phase of buffered (0.1% trifluoroacetic acid) water and acetonitrile. We used a linear gradient from 30% to 80% acetonitrile over 18 min, with an additional 10 min at 100% acetonitrile at the end of the run, with a flow rate of 0.4 mL·min^−1^. Samples were injected in 10 µL volumes and column temperature was maintained at 30 °C. Peaks were identified at 245 nm and integrated by applying the detector response based on peak areas to calibration curves. Concentrations of brominated compounds were expressed as mg·g^−1^ of dry mass of sponge tissue. 

We used the concentration of Chlorophyll *a* (Chl *a*) as an indication on the density of autotrophic symbionts within *A. aerophoba*. Approximately 100 mg of freeze-dried sponge tissue was extracted with 5 mL of 90% acetone for 12 h in the dark at 1 °C. 2 mL of the extract solution were passed through a 0.2 μm PTFE syringe-filter. The 8452A diode array spectrophotometer (Hewlett Packard) was used to perform the absorbance measurements. We measured absorbance at four wavelength (630, 647, 664, and 750 nm) according to the trichromatic equation of Jeffrey and Humphrey [59] to calculate the Chl *a* concentration (1). Chl *a* was expressed as ng·mg^−1^ of dry mass of sponge tissue.


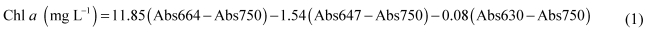


We used several statistical methods from SYSTAT 12 software [60,61] to analyze secondary metabolite abundances and Chl *a* content. Nested analyses of covariance (ANCOVAs) were performed on ranked compound abundances and Chl *a* concentrations with region (2 regions), zone (4 zones), location (8 locations), and sampling site (16 sites) as factors (Figure 1) and depth as a covariate. We used *a posteriori* pairwise comparisons with Bonferroni test to identify the groups responsible for the main significant factors. We also used simple correlation analysis to establish the quantitative relationships between secondary metabolites, Chl *a* and depth.

## 4. Conclusions

The spatial scale explaining the largest variation in the concentration of natural products within a single species depends on the actual compound investigated. Our study showed that the rich secondary chemistry of *Aplysina aerophoba* varied quantitatively from sites less than 500 m apart to geographic regions over 2500 km apart. Aerophobin-1 and Aerophobin-2, and the combined concentration of all BA compounds varied the most between sampling sites less than 500 m apart, while Aplysinamisin-1 and Isofistularin-3 varied across larger spatial scales.

Depth played a trivial role, if any, regulating the concentration of BAs in *A. aerophoba*. Individual correlation analyses with all our data showed that depth could explain a small percentage (8–12%) of the variation in BAs. This minor effect was however overshadowed by the larger influence of spatial scale in the concentrations of BAs. 

The putative role of symbionts on the production of BAs in *A. aerophoba* remains an open question. There is indirect evidence that supports the role of symbionts, including the correlation between Isofistularin-3 and chlorophyll *a* detected in this study. The circumstantial evidence available provides a number of testable hypotheses that, with the use of new molecular techniques, should contribute to the development of this particularly elusive research area.
